# Mineral and Organic Fertilizers Distinctly Affect Fungal Communities in the Crop Rhizosphere

**DOI:** 10.3390/jof8030251

**Published:** 2022-03-01

**Authors:** Mikhail V. Semenov, George S. Krasnov, Vyacheslav M. Semenov, Ariena van Bruggen

**Affiliations:** 1 Department of Soil Biology and Biochemistry, Dokuchaev Soil Science Institute, 119017 Moscow, Russia; 2 Laboratory of Postgenomic Research, Engelhardt Institute of Molecular Biology Russian Academy of Sciences, 119991 Moscow, Russia; gskrasnov@mail.ru; 3 Institute of Physicochemical and Biological Problems in Soil Science of RAS, 142290 Pushchino, Russia; v.m.semenov@mail.ru; 4 Department of Plant Pathology and Emerging Pathogens Institute, University of Florida, Gainesville, FL 32611-0680, USA; ahcvanbruggen@hotmail.com

**Keywords:** fungi, organic fertilizers, NPK, ITS2 rDNA amplicon sequencing, fungal diversity

## Abstract

Fungi represent a diverse group of organisms that play an essential role in maintaining soil health and ecosystem functioning. Plant root exudates form nutrient-rich niches that harbor specific fungal communities, or so-called rhizosphere mycobiomes. The long-term application of fertilizers supplies the soil with nutrients that may override the plant-related effects on rhizosphere fungal communities. Here, we assessed the effect of contrasting fertilization regimes on the composition, diversity, and abundance of bulk soil and rhizosphere mycobiomes of potato, white mustard, and maize under NPK (mineral fertilizers) or fresh cattle manure (organic fertilizers). Mineral and organic fertilizers led to distinct fungal communities in the rhizospheres of all studied crops, and the plant-related effects on the mycobiome were overridden by the effect of fertilization. The abundances of Ascomycota and Olpidiomycota were higher under manure, while the abundances of Basidiomycota and Monoblepharomycota increased under NPK. Manure input strongly increased fungal abundance but decreased fungal diversity and the total number of species. NPK had a slight effect on fungal diversity, but significantly increased the relative abundances of fungal phytopathogens, such as *Alternaria* and *Fusarium*. Our study shows that that potential plant species effects on the abundance and diversity of the rhizosphere mycobiomes are governed by long-term fertilization. Fertilization management could therefore be used to manipulate rhizosphere fungal communities and soilborne pathogen suppressiveness.

## 1. Introduction

Fungi represent a highly diverse group of organisms that play an essential role in maintaining soil health and ecosystem functioning [1]. Fungi are important decomposers and recyclers of recalcitrant or labile organic materials [2]. Although they are often involved in symbiosis with plant roots [3], they can also be soil-borne plant pathogens [4]. Many fungal groups combine these opposite lifestyles—saprophytic, pathogenic, or symbiotic—and they can switch between different strategies depending on the environmental conditions [5]. Despite their high biomass and importance for ecosystem sustainability, the fungal diversity in soil is significantly less studied than the bacterial diversity [4].

Fungi interact with plants at various niches, including the rhizosphere—a narrow zone of soil adjacent to the roots of living plants that is directly influenced by root exudates. Nutrient-rich rhizosphere niches harbor specific fungal communities, including the rhizosphere mycobiome [6]. The rhizosphere mycobiome includes many potential plant pathogens and their antagonists, and can therefore influence plant health and soil disease suppressiveness [7,8]. In the rhizosphere, plant pathogens interact intensively with the rest of the microbial community, which partly determines whether a plant becomes infected [9]. Hence, understanding the factors that shape the composition and intermicrobial relationships of the rhizosphere mycobiome is an important step to control plant health and productivity [10,11].

At a regional level, climate, soil chemistry, and location are usually considered as the major predictors of fungal richness and community composition [12]. Thus, the structure and diversity of soil and rhizosphere mycobiomes are driven by edaphic physicochemical characteristics, such as organic C [13,14], pH [15], and moisture [4]. Soil fungal diversity often is decoupled from plant diversity, although relationships between plant and fungal diversity can be strong locally [4,16,17,18]. The reason for this coupling is that each plant species selects fungal communities in the rhizosphere through the composition of its root exudates [19]. In addition to plant species, the plant developmental stage determines the composition and quantity of rhizodeposits and the associated microbiome [20].

At the farm level, land use and management, such as tillage and fertilization, lead to changes in many soil properties affecting fungal communities [21,22]. Long-term application of mineral fertilizers or fresh farmyard manure supplies large amounts of nutrients to the bulk soil, which is regarded as an oligotrophic environment without these extra nutrients [23]. Introduced nutrients reduce the dependence of the rhizosphere communities on plant-derived C and activate many dormant fungal species. Inorganic N additions may result in increased exudation and soil acidification, changing the soil fungal community [24]. Increased inorganic nutrient availability for plants decreases their dependence on fungal symbioses [25]. Ultimately, inorganic fertilization decreases fungal diversity and biomass [25,26]. In the end, plant-microbe networks in soil often are weakened by the long-term use of inorganic fertilizers [27]. Contrary to inorganic fertilization, the application of organic fertilizer shifts the composition and abundance of fungal communities, and may increase fungal diversity [26,28]. The increase in fungal diversity often leads to root disease suppression [29,30]. Thus, long-term fertilization is a crucial factor determining both rhizosphere nutrient status and fungal communities in agroecosystems. The contribution of long-term fertilization into plant species effects into rhizosphere fungal communities in agroecosystems is not yet fully understood.

This study aimed to evaluate the effect of fertilization- and plant-related (plant species, niches, and plant development stages) factors on the rhizosphere and bulk soil fungal communities in a long-term fertilizer experiment. Specifically, we investigated the differences between mycobiomes in the rhizosphere of crop species when applying high doses of fresh cattle manure or NPK fertilizers. For this purpose, we collected soil and rhizosphere samples of three different crops (potato, white mustard, and maize) from a 7-year fertilizer experiment with three treatments (no fertilization, NPK, and organic cattle manure). Total (DNA) and metabolically active (RNA) fungal communities were analyzed using ITS rDNA metabarcoding and qPCR. We hypothesized that due to the large amounts of nutrients that activate dormant microorganisms, (I) fertilizers would determine both bulk soil and rhizosphere mycobiomes, (II) the effect of plant species would be overridden by the effect of fertilizers in the rhizosphere mycobiomes, and (III) the same type of fertilizers would lead to similar fungal communities in the rhizospheres of different crop species.

## 2. Materials and Methods

### 2.1. Long-Term Microplot Experiment and Soil Sampling

Bulk soil and rhizosphere samples of potato (*Solanum tuberosum* L. var. Zhukovskii), white mustard (*Sinapis alba* L. var. Raduga), and maize (*Zea mays* L Moldavskii 215 MV) were collected in 2017 from a 7-year fertilizer microplot experiment, located in Pushchino, Moscow region, Russia (148.37° E, 34.56° S). The experiment was conducted in compliance with the standards set in the IUCN Policy Statement on Research Involving Species at Risk of Extinction and the Convention on International Trade in Endangered Species of Wild Fauna and Flora. The soil was classified as a Greyzemic Phaeozem Albic [31], characterized by the dominance of fungi in total soil microbial biomass [32] and by balanced microbial growth when adding substrate [33]. The environmental conditions, design of the long-term experiment, and rhizosphere sampling procedure have been described in detail by the authors of [34]. Briefly, fertilizers were evenly distributed over the soil surface and mixed with the soil layer to a depth of 18–20 cm by digging manually. To avoid soil and water movement between plots (0.5 m × 0.5 m), adjacent plots were separated with frames that originally extended about 30 cm into the soil and about 10 cm above the soil surface. In total, 21 experimental plots were constructed: 9 plots with mineral fertilizers, 9 plots with organic fertilizers, and 3 plots without fertilizers and crops (bare fallow). N180 P_2_O_5_150 K_2_O150 (urea, superphosphate, potassium sulfate) or fresh high-fiber cattle manure containing feces mass, urine, and straw at a dose of 50 t ha^−1^ were applied annually once a year in the spring 1 week before sowing crops. Fresh cattle manure had 19.3 ± 0.4% of dry matter with the following composition (per dry matter): total organic carbon (TOC), 35.4 ± 0.5%; total nitrogen (TN), 1.97 ± 0.02%; P_2_O_5_, 1.23 ± 0.03%; K_2_O, 2.06 ± 0.06%. The amounts of nitrogen supplied with cattle manure were almost equivalent to the doses of mineral fertilizers used (190 kg with manure compared to 180 kg N ha^−1^ with mineral fertilizer), while the amounts of potassium were slightly more (192 kg compared to 150 kg K_2_O ha^−1^) and phosphorus were slightly less (136 kg compared to 150 kg P_2_O_5_ ha^−1^). 

Each plant species was grown in 6 plots (3 mineral + 3 organic treatments) in a split-plot design with fertilizer treatments in paired plots and plant species across the paired fertilizer plots. In total, 75 samples were collected (3 crops × 2 fertilizer systems × 2 sample types (soil and rhizosphere) × 2 stages of plant development × 3 replicates + 3 bare fallow treatment replicates). All soil samples were indexed according to the scheme: plant–fertilizer system–soil niche–sampling period (Table S1). Soil samplings were performed on 29 June, 41 days after fertilization (the phase of 2–4 maize leaves, the phase before tuber formation in potatoes, and the phase before flowering in mustard), and 29 July, 71 days after fertilization (the phase of 6–8 maize leaves, the beginning of tuber formation in potatoes, and the beginning of green pods formation in mustard). For all treatments, three replicate soil and rhizosphere samples were collected from each plot. Bulk soil and rhizosphere samples for molecular analyses were stored in sterile bags at −70 °C. Air-dried bulk soil and rhizosphere samples were used for the analysis of chemical soil properties. Microbial biomass carbon was determined in fresh soil samples.

### 2.2. Estimation of Soil Chemical Properties and Microbial Biomass

Soil moisture content was determined gravimetrically by drying soil samples for 24 h at 105 °C. Total organic carbon (TOC) and total nitrogen (TN) contents were determined using a CNS-analyzer Leco 932 (USA). Soil pH was measured with a potentiometer in a 1:2.5 soil/water suspension. Microbial biomass C (MBC) was determined by substrate-induced respiration (SIR) as described previously [35].

### 2.3. Soil DNA and RNA Extraction and Reverse Transcription

The homogenization of soil samples was performed with a Precellys 24 homogenizer (Bertin Technologies, Montigny-le-Bretonneux, France), program 5 (30 s, 6500 revs/min). Total DNA was extracted and purified from 0.25 g of each spatial replicate using the DNeasy PowerSoil Kit (Qiagen, Hilden, Germany). Total RNA was extracted and purified from 2 g of frozen samples using the RNeasy PowerSoil Total RNA Kit (Qiagen, Germany) and Phenol:Chloroform:Isoamyl Alcohol 25:24:1. Co-extracted DNA was removed from RNA samples using RNase-free DNase (Sigma Aldrich, St. Louis, MO, USA). DNA and RNA quality were estimated by electrophoresis in agarose gels (1% *w*/*v* in TAE) with visual detection using the Gel Doc XR+ System (Bio-Rad Laboratories, Hercules, CA, USA). Total RNA was used as the template for cDNA synthesis using the MMLV RT kit (Evrogen Ltd., Moscow, Russia).

### 2.4. Fungal Gene Copies Quantification by qPCR

For each soil sample, 1 µL of DNA from three field replicates was used to quantify the copy numbers of fungal ITS genes. Cloned fragments of *Saccharomyces cerevisiae* Meyen 1B-D1606 ribosomal operons were used to prepare the standard solutions of known concentrations. Gene marker abundance was estimated using EvaGreen Supermix (Bio-Rad, Hercules, CA, USA) and primers ITS1f/5.8S [36]. All reactions were performed in a C1000 Thermal Cycler with the CFX96 Real-Time System (Bio-Rad Laboratories, USA) using the following protocol: 3 min at 95 °C, followed by 49 cycles of 95 °C for 10 s, 50 °C for 10 s, and 72 °C for 20 s. Melting curve analysis was performed to check the amplicon length. 

### 2.5. ITS2 Amplicon Library Sequencing

High-throughput sequencing of the internal transcribed spacer 2 (ITS2) region of the ribosomal RNA encoding genes was performed for 3 interplot replicates of DNA and 1 mixed replicate of cDNA (75 DNA + 25 cDNA). The purified DNA and cDNA isolates were amplified with universal primers gITS7 (5′-GTGARTCATCGARTCTTTG-3′) and ITS4 (5′-TCCTCCGCTTATTGATATGC-3′) [37]. The ITS2 amplicons were generated using the protocol described by the authors of [38]. The sequencing of ITS2 amplicons was performed by Evrogen (Moscow, Russia) on an Illumina MiSeq platform using MiSeq^®^ Reagent Kit v2 (500 cycles) with paired-end 2 × 251 cycle sequencing mode. The raw sequence data were deposited in NCBI SRA under the accession number PRJNA803504.

### 2.6. Bioinformatics and Statistical Analyses

Demultiplexed paired reads were processed in R version 3.5.2 (R Core Team) using the package DADA2 (Divisive Amplicon Denoising Algorithm) as previously described [39]. Briefly, primers were identified and removed using cutadapt [40]. The quality checking, filtering, denoising, and trimming were performed on reads with a maximum of two expected errors per read (maxEE  =  2). The core sample inference algorithm was applied to infer ribosomal sequence variants (RSVs). The forward and reverse reads were merged to obtain the complete denoised sequences, and chimeric RSVs were removed. Taxonomic classification of fungal RSV sequences was performed using the IdTaxa method [41] in the DECIPHER Bioconductor package and the UNITE ITS database v. 8.0 [42]. The negative no-template control was included during PCR amplification. The RSV of *Piloderma* detected in the negative control was removed from the datasets. The non-fungal (Chlorophyta and Cercozoa) sequences were also removed.

Visualization and statistical analyses of the sequencing data were performed using the R packages phyloseq, vegan, ggplot, pheatmap, dendextend, ggdendro, and euler. Alpha-diversity indexes, such as the observed RSV and Shannon index, were calculated. Bray-Curtis dissimilarity was used to explore the variations in fungal community structures among all samples. Non-Metric Multidimensional Scaling (NMDS) was performed on distance matrices to draw 2D graphical outputs. Heatmaps were generated based on the 30 most abundant fungal taxa. To determine the indicator taxa, we considered genera with a share of >0.1%, whose relative abundance increased under the effect of a certain factor by at least 10 times for each of the samples.

The means of three interplot replicates are presented in figures. A multiple *t*-test, as well as Mann-Whitney and Spearman tests, were performed to test for significant (*p* < 0.05) differences of individual fungal taxa among the treatments. To assess the contribution of plant- and fertilizer-related factors to fungal abundances, we calculated Cohen’s d effect size for each factor. The contributions of main and interaction effects among the ecological factors onto dispersion of MBC and fungal gene copy abundances were calculated using the N-way ANOVA test. The differences between soil fungal communities among fertilizer systems, soil niches, crop types, and the stages of plant development, as well as the interactions between these factors, were assessed by Bray-Curtis distance-based permutation tests for homogeneity of multivariate variance (PERMANOVA) and similarities (ANOSIM).

## 3. Results

### 3.1. Rhizosphere and Bulk Soil Chemical Properties

Rhizosphere samples were characterized by significantly (*p* < 0.001) higher TOC, TN, C/N ratios, and pH values than bulk soil samples (Table S1). All of these properties were also significantly (*p* < 0.001) higher in the rhizosphere and bulk soil samples from plots with organic fertilizers than those with mineral fertilizers (Figure S1 and Table S1). The long-term inputs of NPK led to a strong shift in the soil pH in comparison to bare fallow (from 6.1 to 4.33–4.82). The application of manure almost did not change the soil pH (Table S1). The rhizosphere effect on MBC was strongly governed by the fertilizer type and sampling time. Although both plant- and fertilizer-related factors had a positive effect on the MBC values, fertilization made a higher contribution to MBC than the plant-related factors (Figure S1 and Table S2).

### 3.2. Fungal Gene Abundances Estimated by q-PCR

**Figure 1:**
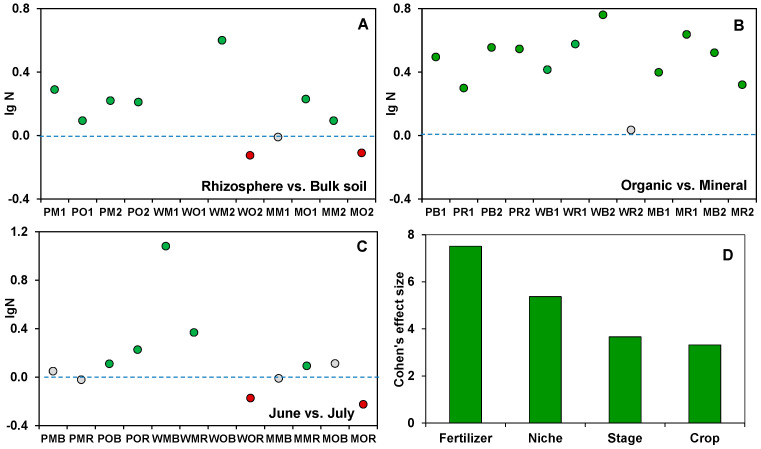
Differences in fungal gene copies abundances between the treatments compared. Comparisons are (**A**) rhizosphere/bulk soil, (**B**) organic/mineral fertilization, and (**C**) two periods of sampling. The green dots indicate significantly higher fungal abundances in soils under compared treatments, red dots indicate significantly lower abundances, and grey dots indicate no statistically significant differences between treatments (*N* = 3). All soil samples are indexed according to the scheme: plant–fertilizer system–soil niche–sampling period. The following indices were used for (I) the crop species: P— potato, W—white mustard, M—maize; (II) fertilizer systems: M—mineral, O—organic; (III) soil niches: B— bulk soil, R—rhizosphere; (IV) periods of sampling: 1—June, 2—July. (**D**) The contribution of the ecological factors (niche, fertilizer system, crop type, stage of plant development) into fungal gene copies abundances estimated using Cohen’s d effect size. The contributions of interaction effects among the ecological factors are presented in Table S2.

The abundances of fungi ranged from 6.76 × 10^9^ to 6.03 × 10^11^ gene copies g-1 soil, and the coefficient of variation was 78%. The sampling time affected the fungal abundance in 7 out of 12 treatments. The differences in fungal abundance between the manure and NPK fertilizer treatments strongly surpassed those between the bulk soil and rhizosphere. A significant rhizosphere effect on fungal gene copy abundances was found in only 9 out of 12 treatments, mostly in plots treated with inorganic fertilizers (Figure 1A). Interestingly, the fungal abundance decreased in the rhizosphere of white mustard and maize under organic fertilization (Figure 1). Each of the considered factors had a significant (*p* < 0.05) effect on the fungal abundance in the rhizosphere and bulk soil. The fertilizer system was characterized by the highest Cohen’s d effect size compared to the plant-related factors (Figure 1). The multifactor variance analysis showed that there were also significant interaction effects of all four factors on fungal abundance (Table S2).

### 3.3. Fungal Community Structure and Composition

In total, 14 fungal phyla (444 genera belonging to 40 classes) were identified. Ascomycota, Basidiomycota, and Mortierellomycota were the dominant phyla within both the total (DNA) and active (RNA) fungal communities (Figure S2 and Table S3). In bulk soil, only the relative abundance of Olpidiomycota was significantly different between the mineral and organic fertilizer systems (Figure S2 and Table S3). In the rhizosphere, the abundances of Ascomycota and Olpidiomycota were higher under manure (*p* < 0.01), while the abundances of Basidiomycota and Monoblepharomycota were higher under NPK (*p* < 0.01) (Table S3). At the second sampling time, the shares of Mortierellomycota, Chytridiomycota, and Glomeromycota were lower (*p* < 0.05) under white mustard, and the share of Mortierellomycota was lower under potato.

**Figure 2:**
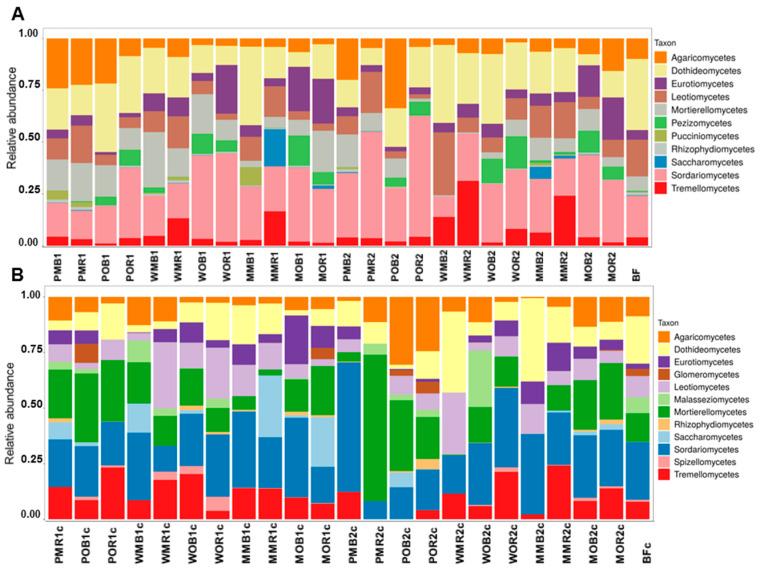
The structure of total (**A**) and active (**B**) fungal communities in the studied samples at the class level (the data are presented for phyla with abundances over 0.5%). All soil samples are indexed according to the scheme: plant–fertilizer system–soil niche–sampling period. The following indices were used for (I) the crop species: P—potato, W—white mustard, M—maize; (II) fertilizer systems: M—mineral, O—organic; (III) soil niches: B—bulk soil, R—rhizosphere; (IV) periods of sampling: 1—June, 2—July. The bare fallow is marked as BF. Acronyms of the RNA-based values have “c” indexes in titles.

At the class level, *Agaricomycetes*, *Dothideomycetes*, *Leotiomycetes*, and *Tremellomycetes* were the dominant taxa in unfertilized soil (treatment BF). *Geminibasidiomycetes* and *Glomeromycetes* were the only two classes characterized by the higher relative abundances under BF treatment compared to all fertilized soils (Figure 2). Soils under long-term fertilization had higher relative abundances of *Rhizophydiomycetes* (both with NPK and manure) and *Pezizomycetes* (only with manure) compared to unfertilized soil. *Saccharomycetes* were found only under treatments with maize. *Eurotiomycetes* were also associated with maize, but only under manure. *Tremellomycetes* had higher abundances under mustard and maize with NPK. *Agaricomycetes* were associated with potatoes (Figure 2). The contributions of Mortierellomycetes and Tremellomycetes to active mycobiomes were higher compared to total communities, while the relative abundances of *Dothediomycetes* and *Rhizophydiomycetes* were higher in total communities compared to active ones. High relative abundances of *Agaricomycetes*, *Leotiomycetes*, and *Sordariomycetes* were revealed in most treatments for both total and active mycobiomes.

**Figure 3:**
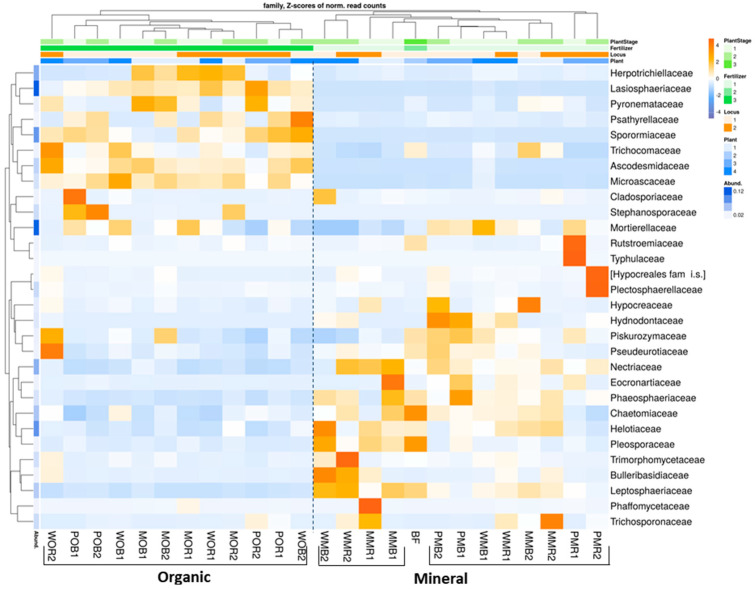
Relative abundances of top 30 fungal taxa in total (DNA) fungal communities of the considered treatments at the family level (*N* = 3). The data are given as Z-scores. Abundances of fungal classes with positive Z-scores are marked with orange color, abundances with negative z-scores are marked with blue color. All soil samples are indexed according to the scheme: plant–fertilizer system–soil niche–sampling period. The following indices were used for (I) the crop species: P— potato, W—white mustard, M—maize; (II) fertilizer systems: M—mineral, O—organic; (III) soil niches: B— bulk soil, R—rhizosphere; (IV) periods of sampling: 1—June, 2—July. The bare fallow without fertilizers is marked as BF.

At the family level, bulk soil and rhizosphere mycobiomes were more clearly distinguished in the organic fertilizer plots compared to the NPK-treated plots (Figure 3). Mycobyomes of bare fallow soil were similar to fungal communities under the long-term application of NPK. The families *Ascodesmidaceae*, *Herpotrichiellaceae*, *Lasiosphaeriaceae*, *Microascaceae*, *Psathyrellaceae*, *Pyronemataceae*, *Sporormiaceaea*, and *Trichocomaceae* became dominant under organic fertilization (Figure 3). On the other hand, long-term application of manure led to decreases in the relative abundances of many other fungal taxa, namely *Nectriaceae*, *Piskurozymaceae*, *Pseudeurotiaceae*, *Phaeosphaeraceae*, *Chaetomiaceae*, *Helotiaceae*, *Pleosporaceae*, and *Leptosphaeriaceae* (Figure 3). Compared to organic fertilization, the application of NPK increased the abundances of *Eocronartiaceae*, *Hypocreaceae*, *Hydnodontaceae*, and *Nectriaceae*. Both fertilizer systems increased the relative abundance of *Mortierellaceae* and decreased the abundance of *Rutstroemiaceae* compared to fallow soil. The plant-related effects were not significant for most fungal families. However, *Plectosphaerellaceae*, *Rutstroemiaceae*, and *Typhulaceae* increased only in the potato rhizosphere under NPK. Similarly, *Bulleribasidiaceae* and *Trimorphomycetaceae* developed only under white mustard with NPK.

**Figure 4:**
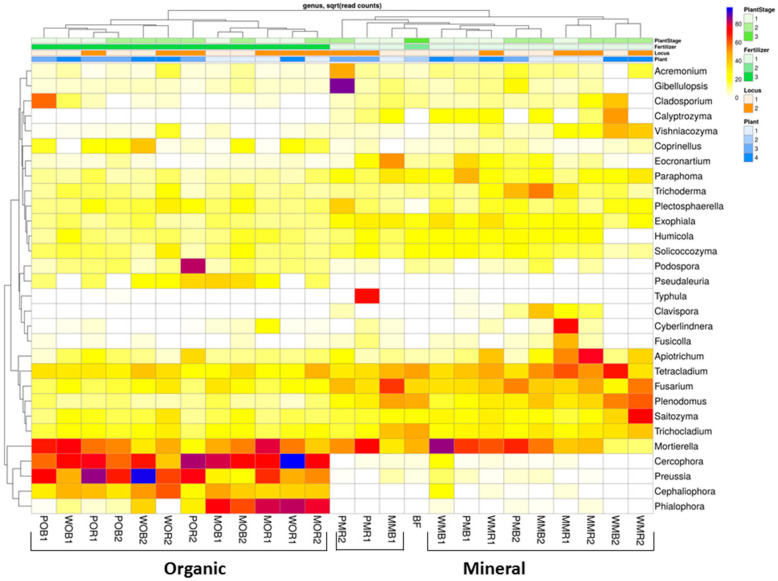
Relative abundances of top 30 fungal taxa in the total (DNA) fungal communities of the considered treatments at the genera level (*N* = 3). The data are given as square roots of the read counts. Abundances of fungal genera increase in a row: white–yellow–red–violet–dark blue. All soil samples are indexed according to the scheme: plant–fertilizer system–soil niche–sampling period. The following indices were used for (I) the crop species: P—potato, W—white mustard, M—maize; (II) fertilizer systems: M—mineral, O—organic; (III) soil niches: B—bulk soil, R—rhizosphere; (IV) periods of sampling: 1—June, 2—July. The bare fallow without fertilizers is marked as BF.

At the genera level, both bulk soil and rhizosphere mycobiomes were similar to each other, but the mycobiomes could be separated according to fertilizer system type (Figure 4). When organic fertilizers were applied, the structures of the fungal total and active communities underwent a significant restructuring in the rhizosphere and bulk soil: The relative abundances of *Cephaliophora*, *Cercophora*, *Phialophora*, and *Preussia* were significantly increased in manured plots compared to the communities in NPK plots (Figure S3; Table S4). Mineral fertilizers changed the soil fungal communities only slightly compared to BF, and resulted in the dominance of *Apiotrichum*, *Calyptrozyma*, *Fusicola*, and *Fusarium* (Figure 4). There were significant mycobiome differences between organic and mineral fertilizer treatments regardless of the niche (rhizosphere or bulk soil). The abundances of *Mortierella* and *Plectosphaerella* increased under the mineral and organic fertilizer systems compared to the unfertilized control, except for plots that were planted with white mustard.

The dominant genera—*Preussia*, *Fusarium*, *Gibberella*, and *Exophiala*—had similar relative abundances in total and active mycobiomes. However, the abundances of many other fungal genera differed in the RNA database from those in the DNA dataset. In the DNA dataset, *Cephaliophora* and *Cercophora* had higher relative abundances in soils with manure compared to the genera in the RNA dataset, while shares of *Eocronartium*, *Paraphoma*, and *Rhizophydium* were higher in soils with NPK (Table S4). On the contrary, *Malassezia*, *Mortierella*, *Mycosphaerella*, *Saitozyma*, *Solicoccozyma*, *Tetracladium*, and *Trichoderma* were more abundant in active mycobiome than those in total communities for both fertilization systems.

### 3.4. Indicator Taxa

A total of 25 genera of fungi were associated with the rhizosphere of a particular crop (Table S5). Among most numerous (>1%) genera, *Clonostachys* (1.3% in June, 4.6% in July under NPK), *Colletotrichum* (low in June, up to 2% in July), *Gibellulopsis* (low in June, up to 34.4% in July under NPK), and *Podospora* (low in June, up to 20.5% in July under manure) were clearly indicator taxa in the potato rhizosphere. *Conocybe* (1% in June, 0.37% in July under manure) and *Cyberlindnera* (15.1% under NPK or 1% under manure in June, low in July) were indicator taxa with a relative abundance of >1% in the maize rhizosphere. Another 21 genera were indicator taxa for two of the three plant species (Table S5). For instance, the genus *Phialophora* was detected as an indicator taxon with a high relative abundance in both maize and white mustard rhizospheres (up to 39% for mustard under manure). 

The rhizosphere mycobiome underwent significant changes under the influence of fertilizers, but not crop type or niche. Of the 106 fungal genera detected, 24% changed their share in the community at least by an order of magnitude in response to different fertilizers (Table S6). Most of the rhizospheric indicator taxa were fertilizer-related (Table S6). These genera were divided into five groups. Specifically, 27 genera responded positively to the application of NPK, 22 genera were associated with the application of manure, and 21 genera responded positively to both fertilizer systems (Table S6). As a result of long-term manure application, 30 fungal genera were not detected in these communities, while only 7 genera were suppressed by NPK inputs.

### 3.5. Potential Pathogenic Genera

**Figure 5:**
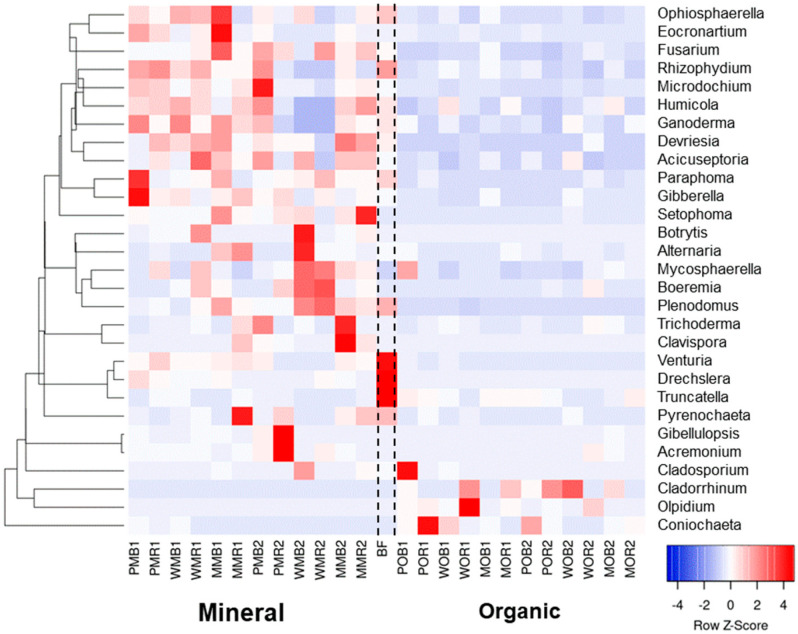
Relative abundances of major fungal plant pathogens plus biocontrol genera *Cladorrhinum*, *Humicola*, and *Trichoderma* in the total (DNA) fungal communities of the considered treatments at the genera level (*N* = 3). The data are given as Z-scores. Abundances of fungal genera with positive z-scores are marked with red color, abundances with negative z-scores are marked with blue color. All soil samples are indexed according to the scheme: plant–fertilizer system–soil niche–sampling period. The following indices were used for (I) the crop species: P—potato, W—white mustard, M—maize; (II) fertilizer systems: M—mineral, O—organic; (III) soil niches: B—bulk soil, R—rhizosphere; (IV) periods of sampling: 1—June, 2—July. The bare fallow without fertilizers is marked as BF.

We performed additional analyses on potentially plant pathogenic fungal taxa that were abundant (Figure 5 and Table S4). We also considered the genera *Trichoderma, Cladorrhinum*, and *Humicola*, which are known as biocontrol agents against plant pathogens and opportunistic avirulent plant symbionts. Among the genera considered, five potentially plant pathogenic taxa were present in large quantities (>1%) in unfertilized bare fallow soil: *Drechslera* (4.2%), *Fusarium* (3.7%), *Paraphoma* (2.2%), *Plenodomus* (6.8%), and *Venturia* (1.5%). Both fertilizer systems decreased the relative abundances of *Drechslera* and *Venturia* in bulk soil and rhizospheres compared to bare fallow (Figure 5). However, NPK application led to a strong increase in the relative abundances of most potentially pathogenic genera except *Ganoderma*, *Paraphoma*, *Pyrenochaeta*, and *Truncatella* (Figure 5 and Table S4). Under NPK, *Gibellulopsis* increased up to 23%; *Fusarium* and *Gibberella* (the teleomorph of some *Fusarium* species) increased up to 12.7% and 3%, respectively; *Eocronartium* increased up to 8.0%; and *Plenodomus* increased up to 10.7%, depending on the crops grown (Figure 5). Conversely, bulk soil and rhizosphere samples under manure were characterized by very low relative abundances of most fungal genera detected. Only 3 of 26 genera were exceptions (*Cladosporium*, *Coniochaeta*, and *Olpidium*), although they were in a low relative abundance (less than 0.5%). Potentially antagonistic genera *Humicola* and *Trichoderma* increased their abundance in soils with NPK, while the share of *Cladorrhinum* was higher under organic fertilization. Together, the plant pathogenic genera had a median relative abundance of 22.7% in NPK plots and 3.9% in manure plots.

### 3.6. α-Diversity

**Figure 6:**
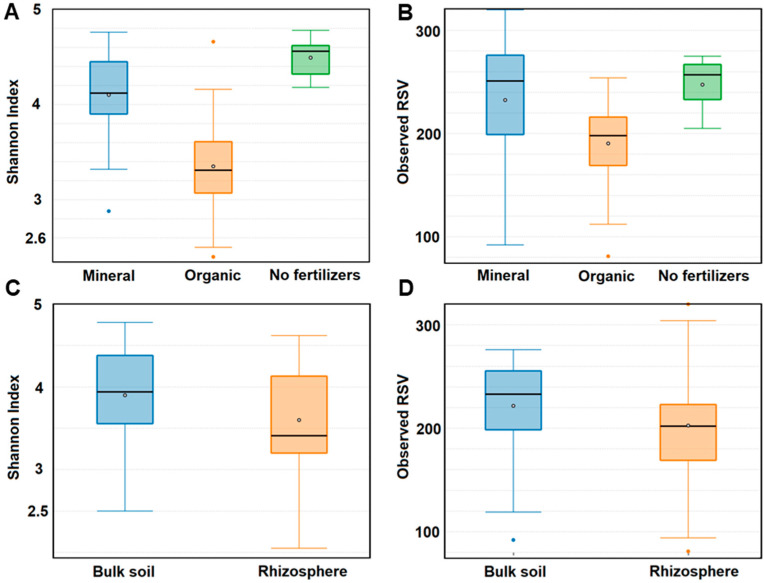
Alpha-diversity of bulk soil and rhizosphere mycobiomes. (**A**,**B**) Numbers of ribosomal sequence variants (RSV) and Shannon indexes when comparing all samples corresponding to mineral and organic fertilizer system (*N* = 36). (**C**,**D**) Numbers of RSV and Shannon indexes when comparing all rhizosphere and bulk soil samples (*N* = 36).

Long-term application of mineral fertilizers almost did not affect the number of fungal genera and RSVs in soils compared to bare fallow without fertilizers. The genera and RSV numbers of fungi decreased under the application of fresh manure by 40% and 20%, respectively (Figure 6B). There was a significant decrease of Shannon indexes (from 4.5 to 3.4) in manure plots, and a slight decrease to 4.1 in mineral fertilizer plots (Figure 6A). The numbers of fungal RSVs and Shannon indexes were higher in the bulk soil than in the rhizosphere (Figure 6C,D).

### 3.7. β-Diversity

**Figure 7:**
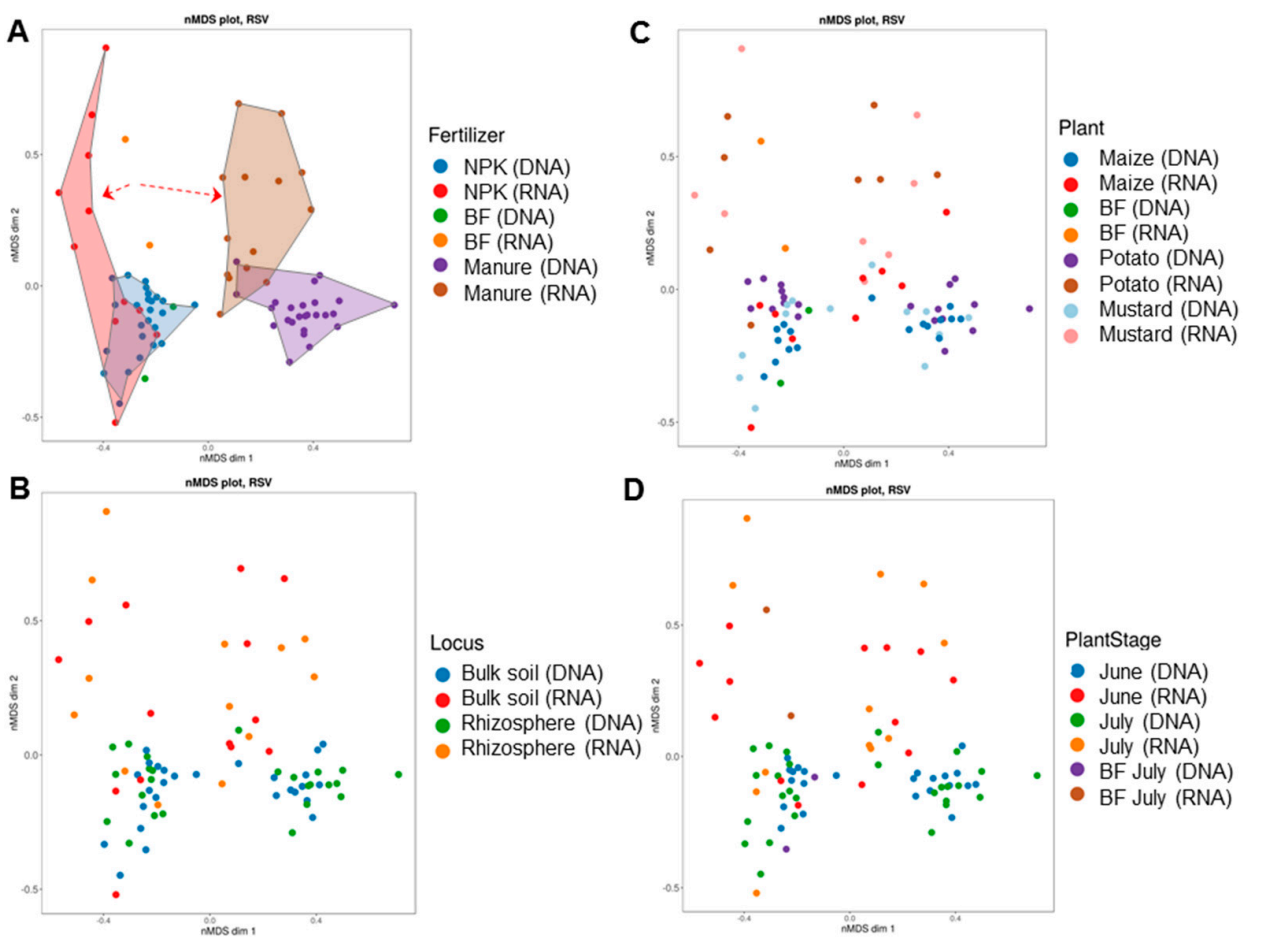
Non-metric multidimensional scaling plots of soil fungal community assembly patterns using the Bray-Curtis (BC) distance matrix as related to (**A**) fertilizer system, (**B**) soil niche, (**C**) crop type, and (**D**) stage of plant development. The dotted arrows indicate the divergence of the bulk soil and rhizosphere mycobiomes under NPK and manure in opposite directions from the control without fertilizers.

All mycobiomes were clustered into four groups based on Bray-Curtis metrics (Figure 7). The clusters were distributed along two axes, corresponding to the type of fertilizer applied (NPK or manure) and the metabolic state (total or active) of the mycobiomes (Figure 7A). Fungal communities of soil under bare fallow were clustered within the mineral fertilization clusters and separately from manure clusters. Factors associated with plants (crop type, stage of plant development, and soil niche) could not explain the differences between fungal communities (Figure 7B–D and Table S7). Almost all differences between mycobiomes were caused by fertilization (*F* = 12.98, *p* = 0.001; *R* = 0.877, *p* = 0.001). The influence of the plant species on communities’ dissimilarity was statistically significant. However, the size of the effect was small (*F* = 1.72, *p* = 0.026; *R* = 0.053; *p* = 0.084) compared to that of fertilization (Table S7). No individual significant effects of the soil niche and stage of plant development on the β-diversity of the mycobiome structures were revealed (Table S7). The fertilizer system alone had a higher effect on dissimilarities in the fungal communities than the interactions between the considered factors.

## 4. Discussion

### 4.1. Long-Term Organic Fertilization Shapes Rhizosphere and Bulk Soil Mycobiome and Reduces Its Diversity

The conducted study shows that fertilization can be a crucial factor determining the structure, diversity, and abundance of fungal communities in bulk soil and plant rhizosphere. We identified two groups of fungal communities: (1) bulk soil and rhizosphere under NPK or without fertilization, and (2) bulk soil and rhizosphere under manure. Despite the sharp decline in soil pH due to the long-term application of physiologically acidic mineral fertilizers, fungal communities under NPK treatment did not differ significantly from those in unfertilized soils. In turn, the application of organic fertilizers hardly changed the soil pH but significantly increased the microbial biomass and fungal abundance. In addition, the application of organic fertilizers decreased the fungal diversity and prevented the detection of many fungal taxa from the bulk soil and rhizosphere. Thus, our study confirms previous findings that soil fungal community composition is primarily driven by total organic carbon content rather than soil pH [13,14].

Nevertheless, the effect of fertilizer systems on the soil fungal communities is not yet fully understood. Organic fertilization commonly increases fungal abundance in soils with manure [14,28], but NPK application could increase [43] or decrease it [28]. Similarly, fungal diversity may decrease [26,43] or remain unchanged [24,28,44,45] under long-term mineral fertilization. Some authors have explained this variation by the difference in pH across soils. Long-term mineral fertilization decreases fungal diversity in neutral soils (pH > 6, such as Phaeozems in our study) rather than in acidic soils (pH < 6) [28,45]. Trends in fungal diversity under organic fertilizers are even more variable. The application of manure may increase fungal diversity [26,28] or decrease it [44], or it may remain unchanged [45].

Among the most abundant fungal genera, *Mortierella* was the only taxon that increased its abundance under both mineral and organic fertilizer systems. Species of *Mortierella* live as saprotrophs in soil and are usually non-pathogenic for plants. *Mortierella* could also promote plant growth and are dominant in suppressive soils [46]. The application of mineral fertilizers increased the abundance of many phytopathogenic taxa, particularly *Fusarium*. Under organic fertilization, *Cephaliophora*, *Cercophora*, *Phialophora*, and *Preussia* became the most represented genera in bulk soil and rhizosphere mycobiomes. *Cephaliophora* consists of rotifer-capturing species, while *Cercophora* are typical coprophilous species [47]. The genus *Phialophora* was also detected by plating on solid media in our previous study, and was identified as *Phialophora fastigiata*—a saprophyte commonly found in soil or on decaying wood [48]. Several *Preussia* species produce bioactive secondary metabolites, particularly the preussomerins, which perform an antimicrobial activity [49].

Plate counting on Czapec and PDA media gave the opposite results for cultivated fungal diversity in the same treatments as studied here: Applications of NPK led to a decrease in cultivated fungal diversity, while organic fertilization increased it [48]. Altogether, 39 fungal species belonging to 19 genera were cultivated. This is less than 5% of the total fungal diversity (444 genera) obtained by DNA metabarcoding in this study. However, some fungal genera detected by plating are not always found by DNA metabarcoding, e.g., *Epicoccum* and *Sarocladium*. *Penicillium* was the dominant genus in cultivated communities [48]. However, it was a minority taxon in our current experiment. In contrast to our current results, the abundance of cultivated *Trichoderma* was higher in manure plots than in soils treated with NPK [48]. Thus, the results based on culture-dependent and -independent techniques using the same soil samples may be completely different and may sometimes arrive at opposite conclusions. The differences may be due to the increased detection of sporulating fungi by cultivation on solid media and the decreased detection of these fungi by direct DNA or RNA extraction and metabarcoding as it is more difficult to extract nucleic acids from spores than from mycelium.

### 4.2. Long-Term Fertilization Overrides Plant Species Effects on Rhizosphere Mycobiomes

The effect of fertilizers on fungal communities was detected not only in the bulk soil but also in the rhizosphere of the studied crop species. Moreover, the influence of plant-related factors on rhizosphere mycobiomes was much less compared to long-term fertilization. The plant-related rhizosphere effect was indicated by an increase in fungal abundance and biomass, as well as in the dominance of some fungal indicator taxa. However, this effect was governed by what type of fertilizer was applied—mineral or organic. 

Plants and fungi often have strong interspecies relationships. Therefore, rhizosphere mycobiomes are commonly considered to differ from those in bulk soil and between plant species [50]. Fungi are heterotrophic organisms that depend on exogenous C for growth, and plant root exudates contain C substrates for their growth and development. We revealed that fertilization, regardless of the type, led to the convergence of the rhizosphere and bulk soil fungal community structures. This is in accordance with our previous culture-dependent analysis [48]. Long-term input of mineral or organic fertilizers may decrease a preference of the rhizosphere microbiome for root-derived substrates [51], since soil labile organic carbon introduced with organic fertilization is more than enough to level out the contribution of root exudates [34]. The long-term application of mineral fertilizers without additional C source also alters the rhizosphere mycobiome [24] and weakens plant-microbe networks [27].

On the other hand, the effect of fertilizers on the fungal community of the soil versus the rhizosphere was much lower than on bacteria [52,53]. First, fungi are less sensitive to changes in pH caused by the application of mineral fertilizers. Second, the plant-related effect was much higher for the rhizosphere mycobiome in the NPK plots: The interaction between the plant species and its stage of development had a strong impact on the fungal abundance. Unlike bacteria, many indicative fungal taxa were associated with a particular plant species.

### 4.3. Plant Pathogens in Rhizosphere and Soil Suppressiveness Management

Long-term application of manure drastically reduced the soil and rhizosphere fungal diversity in our study. Microbial diversity is considered as one of the factors responsible for the sustainable functioning of soil systems [54,55]. Rhizosphere microbiomes influence the productivity of plant communities, promote plant growth, and protect plants from pathogens [7]. The latter is closely related to soil suppressive activity against plant pathogens [8,56]. Higher microbial diversity in the rhizosphere of plants has been shown to increase soil suppressiveness [46].

Indeed, organic fertilization significantly reduced the relative abundances of most pathogenic genera detected in our study. A similar effect of organic fertilizers was found in previous studies, e.g., for *Alternaria*, *Fusarium*, and *Gibberella* [15,28,44]. Long-term application of manure also increased the relative abundance of *Cladorrhinum*, a potential biological control agent for the reduction of *Rhizoctonia solani* [57]. Organic amendments have been proposed as a strategy for the management of plant diseases caused by soil-borne pathogens [29,56], and they have been effective in the suppression of *Fusarium* [58], *Verticillium* [30], *Pyrenochaeta* [59], and many other fungal genera [29]. The suppressive effect is likely related to an increase of pathogen-antagonistic fungi (e.g., *Mortierella*, *Pseudaleuria*, and *Hypocreales* [28,46]) or biocontrol bacteria, such as *Collimonas* and *Lysobacter* [51], since the organic fertilizers may act as an alternative C source for the antagonists. Banerjee et al. also showed that the network connectivity and abundance of keystone microbial taxa were higher under organic farming than conventional farming, which could be related to higher soil suppressiveness [60].

Contrary to the suppressive effects of organic fertilization, the long-term application of mineral fertilizers led to an increase in the relative abundances of several potential phytopathogenic genera, such as *Alternaria*, *Gibellulopsis*, *Fusarium*, *Gibberella*, *Eocronartium*, and *Plenodomus*. In some samples, the sum of potential plant pathogenic genera reached more than 40% of the total mycobiome. Increases in phytopathogens [15,24,26] and root diseases were shown to be associated with mineral fertilizer applications in previous studies [55,61].

The abundances of *Trichoderma* and *Humicola* also increased with mineral fertilizers in our experiment. *Trichoderma* species are opportunistic, avirulent plant symbionts, which can be parasites and antagonists of many phytopathogenic fungi, thus protecting plants from disease [62]. *Humicola* species from soil are considered as potential antagonists for the biological control of plant diseases [63]. However, the high abundances of *Trichoderma* and *Humicola* were associated with large numbers of phytopathogenic fungi. Therefore, antagonistic activity related to phytopathogens depends on specific ecological conditions and may not necessarily occur. Moreover, the antagonistic effect may not occur at the same time, but at a lag that can be observed only by sampling frequently over time.

The large differences between the effects of organic and mineral fertilization on fungal communities are likely partially due to the differences in the soil food web. Fertilization by manure likely strengthens the microbe-eukaryote associations, such as survival, predation, and cooperation, more than NPK application [64]. Fungal mycelium is an efficient nutrient source for soil nematodes, collembola, oribatid mites, and enchytraeids due to the low C/N ratios of fungal cords and hyphae compared to plant-derived organic matter [65]. Since soil fauna can suppress the abundance of many plant pathogenic fungi (e.g., *Fusarium* spp.) in agricultural ecosystems [66], organic amendments might therefore be used to control the soil-borne phytopathogens by manipulating the structure of detrital food webs.

## 5. Conclusions

In summary, our findings suggest that fertilization has a crucial impact on the rhizosphere, bulk soil total, and active mycobiomes, determining the fungal community structure, abundance, and diversity. In addition, fertilization affects the growth of individual species of plant pathogens and their antagonists. Plant species effects on the rhizosphere and bulk soil mycobiome were governed by the introduction of NPK or fresh farmyard manure. Organic fertilizers sharply increased microbial biomass and fungal abundance, and suppressed fungal phytopathogens. Conversely, mineral fertilization stimulated the abundance of pathogens in the bulk soil and rhizosphere. Organic amendments may therefore be used to manipulate native rhizosphere fungal communities and enhance soil suppressiveness against plant pathogens. The effectiveness of manure amendments, however, would be strongly dependent on its quality and fiber content, as well as the duration and technology of manure storage. Controlling the soil animal food webs by manure amendments is also of high importance to reduce the abundance soil-borne fungal phytopathogens. Understanding how a soil becomes suppressive, as well as which microorganisms and associated food webs provide a suppressive effect, will allow us to engineer the soil microbiome to enhance plant health.

## Data Availability

The raw sequence data were deposited in NCBI SRA under the accession number PRJNA803504.

## Supplementary Materials

The following supporting information can be downloaded at: https://www.mdpi.com/article/10.3390/jof8030251/s1, Figure S1: (A) Microbial biomass carbon (MBC) of the studied samples. (B) The contribution of the ecological factors (niche, fertilizer system, crop type, stage of plant development) into MBC estimated using Cohen’s d effect size; Figure S2: The structure of total (upper) and active (lower) fungal communities in studied samples at phylum level; Figure S3: The structure of total (upper) and active (lower) fungal communities in studied samples at genera level; Table S1: Basic chemical characteristics of the studied soil and rhizosphere samples; Table S2: The contribution of main and interaction effects among the ecological factors (niche, fertilizer system, crop type, stage of plant development) onto dispersion of MBC and fungal gene copies; Table S3: The lack of overall response of the most abundant fungal phyla to fertilization (Organic vs. Mineral) in bulk soil and rhizosphere; Table S4: The top 70 fungal genera responsive to fertilization (Organic vs. Mineral) in bulk soil and rhizosphere; Table S5: Fungal taxa associated with rhizosphere of potato, white mustard, and maize.; Table S6: Fungal taxa associated with organic or mineral fertilizers; Table S7: PERMANOVA and ANOSIM tests of variance and similarities of total fungal communities’ using Bray-Curtis (BC) distance matrix.

## References

[1] van Bruggen A.H., Goss E.M., Havelaar A., van Diepeningen A.D., Finckh M.R., Morris J.G. (2019). One Health-Cycling of diverse microbial communities as a connecting force for soil, plant, animal, human and ecosystem health. Sci. Total Environ..

[2] Treseder K.K., Lennon J.T. (2015). Fungal traits that drive ecosystem dynamics on land. Microbiol. Mol. Biol. Rev..

[3] Bennett J.A., Maherali H., Reinhart K.O., Lekberg Y., Hart M.M., Klironomos J. (2017). Plant-soil feedbacks and mycorrhizal type influence temperate forest population dynamics. Science.

[4] Tedersoo L., Bahram M., Põlme S., Kõljalg U., Yorou N.S., Wijesundera R., Ruiz L.V., Vasco-Palacios A.M., Thu P.Q., Suija A. (2014). Global diversity and geography of soil fungi. Science.

[5] Zanne A.E., Abarenkov K., Afkhami M.E., Aguilar-Trigueros C.A., Bates S., Bhatnagar J.M., Flores-Moreno H., Floudas D., Gazis R., Hibbett D. (2020). Fungal functional ecology: Bringing a trait-based approach to plant-associated fungi. Biol. Rev..

[6] Pagano M.C., Correa E.J.A., Duarte N.F., Yelikbayev B., O’Donovan A., Gupta V.K. (2017). Advances in eco-efficient agriculture: The plant-soil mycobiome. Agriculture.

[7] Raaijmakers J.M., Paulitz T.C., Steinberg C., Alabouvette C., Moënne-Loccoz Y. (2009). The rhizosphere: A playground and battlefield for soilborne pathogens and beneficial microorganisms. Plant Soil.

[8] Mendes R., Garbeva P., Raaijmakers J.M. (2013). The rhizosphere microbiome: Significance of plant beneficial, plant pathogenic, and human pathogenic microorganisms. FEMS Microbiol. Rev..

[9] Chaparro J.M., Sheflin A.M., Manter D.K., Vivanco J.M. (2012). Manipulating the soil microbiome to increase soil health and plant fertility. Biol. Fertil. Soils.

[10] Mueller U.G., Sachs J.L. (2015). Engineering microbiomes to improve plant and animal health. Trends Microbiol..

[11] Raaijmakers J.M., Mazzola M. (2016). Soil immune responses. Science.

[12] Nilsson R.H., Anslan S., Bahram M., Wurzbacher C., Baldrian P., Tedersoo L. (2019). Mycobiome diversity: High-throughput sequencing and identification of fungi. Nat. Rev. Microbiol..

[13] Rousk J., Bååth E., Brookes P.C., Lauber C.L., Lozupone C., Caporaso J.G., Knight R., Fierer N. (2010). Soil bacterial and fungal communities across a pH gradient in an arable soil. ISME J..

[14] Liu J., Sui Y., Yu Z., Shi Y., Chu H., Jin J., Liu X., Wang G. (2015). Soil carbon content drives the biogeographical distribution of fungal communities in the black soil zone of northeast China. Soil Biol. Biochem..

[15] Hu X., Liu J., Wei D., Zhu P., Cui X.A., Zhou B., Chen X., Jin J., Liu X., Wang G. (2017). Effects of over 30-year of different fertilization regimes on fungal community compositions in the black soils of northeast China. Agric. Ecosyst. Environ..

[16] Nguyen N.H., Williams L.J., Vincent J.B., Stefanski A., Cavender-Bares J., Messier C., Paquette P., Gravel D., Reich P.B., Kennedy P.G. (2016). Ectomycorrhizal fungal diversity and saprotrophic fungal diversity are linked to different tree community attributes in a field-based tree experiment. Mol. Ecol..

[17] Prober S.M., Leff J.W., Bates S.T., Borer E.T., Firn J., Harpole W.S., Lind E.M., Seabloom E.W., Adler P.B., Bakker J.D. (2015). Plant diversity predicts beta but not alpha diversity of soil microbes across grasslands worldwide. Ecol. Lett..

[18] Tedersoo L., Bahram M., Cajthaml T., Põlme S., Hiiesalu I., Anslan S., Harend H., Buegger F., Pritsch K., Koricheva J. (2016). Tree diversity and species identity effects on soil fungi, protists and animals are context dependent. ISME J..

[19] Zeilinger S., Gupta V.K., Dahms T.E., Silva R.N., Singh H.B., Upadhyay R.S., Gomes E.V., Tsui C.K.-M., Nayak S.C. (2016). Friends or foes? Emerging insights from fungal interactions with plants. FEMS Microbiol. Rev..

[20] Chaparro J.M., Badri D.V., Vivanco J.M. (2014). Rhizosphere microbiome assemblage is affected by plant development. ISME J..

[21] Essel E., Xie J., Deng C., Peng Z., Wang J., Shen J., Xie J., Coulter J.A., Li L. (2019). Bacterial and fungal diversity in rhizosphere and bulk soil under different long-term tillage and cereal/legume rotation. Soil Tillage Res..

[22] Pan H., Chen M., Feng H., Wei M., Song F., Lou Y., Cui X., Wang H., Zhuge Y. (2020). Organic and inorganic fertilizers respectively drive bacterial and fungal community compositions in a fluvo-aquic soil in northern China. Soil Tillage Res..

[23] Blagodatskaya E., Kuzyakov Y. (2013). Active microorganisms in soil: Critical review of estimation criteria and approaches. Soil Biol. Biochem..

[24] Paungfoo-Lonhienne C., Yeoh Y.K., Kasinadhuni N.R.P., Lonhienne T.G., Robinson N., Hugenholtz P., Ragan M.A., Schmidt S. (2015). Nitrogen fertilizer dose alters fungal communities in sugarcane soil and rhizosphere. Sci. Rep..

[25] Lin X., Feng Y., Zhang H., Chen R., Wang J., Zhang J., Chu H. (2012). Long-term balanced fertilization decreases arbuscular mycorrhizal fungal diversity in an arable soil in North China revealed by 454 pyrosequencing. Environ. Sci. Technol..

[26] Ding J., Jiang X., Guan D., Zhao B., Ma M., Zhou B., Cao F., Yang X., Li L., Li J. (2017). Influence of inorganic fertilizer and organic manure application on fungal communities in a long-term field experiment of Chinese Mollisols. Appl. Soil Ecol..

[27] Huang R., McGrath S.P., Hirsch P.R., Clark I.M., Storkey J., Wu L., Zhou J., Liang Y. (2019). Plant–microbe networks in soil are weakened by century-long use of inorganic fertilizers. Microb. Biotechnol..

[28] Xiang X., Liu J., Zhang J., Li D., Xu C., Kuzyakov Y. (2020). Divergence in fungal abundance and community structure between soils under long-term mineral and organic fertilization. Soil TillageRes..

[29] Bonanomi G., Antignani V., Capodilupo M., Scala F. (2010). Identifying the characteristics of organic soil amendments that suppress soilborne plant diseases. Soil Biol. Biochem..

[30] Lang J., Hu J., Ran W., Xu Y., Shen Q. (2012). Control of cotton Verticillium wilt and fungal diversity of rhizosphere soils by bio-organic fertilizer. Biol. Fertil. Soils.

[31] IUSS Working Group (2015). WRB World Reference Base for Soil Resources 2014. International Soil Classification System for Naming Soils and Creating Legends for Soil Maps.

[32] Loeppmann S., Semenov M., Kuzyakov Y., Blagodatskaya E. (2018). Shift from dormancy to microbial growth revealed by RNA: DNA ratio. Ecol. Indic..

[33] Semenov M.V., Stolnikova E.V., Ananyeva N.D., Ivashchenko K.V. (2013). Structure of the microbial community in soil catena of the right bank of the Oka River. Biol. Bull..

[34] Semenov M.V., Krasnov G.S., Semenov V.M., van Bruggen A.H. (2020). Long-term fertilization rather than plant species shapes rhizosphere and bulk soil prokaryotic communities in agroecosystems. Appl. Soil Ecol..

[35] Semenov M., Blagodatskaya E., Stepanov A., Kuzyakov Y. (2018). DNA-based determination of soil microbial biomass in alkaline and carbonaceous soils of semi-arid climate. J. Arid Environ..

[36] Fierer N., Jackson J.A., Vilgalys R., Jacksson R.B. (2005). Assessment of soil microbial community structure by use of taxon-specific quantitative PCR assays. Appl. Environ. Microb..

[37] Ihrmark K., Bödeker I.T.M., Cruz-Martinez K., Friberg H., Kubartova A., Schenck J., Strid Y., Stenlid J., Brandström-Durling M., Clemmensen K.E. (2012). New primers to amplify the fungal ITS2 region–evaluation by 454-sequencing of artificial and natural communities. FEMS Microbiol. Ecol..

[38] Lear G., Dickie I., Banks J., Boyer S., Buckley H.L., Buckley T., Cruickshank R., Dopheide A., Handley K.M., Hermans S. (2018). Methods for the extraction, storage, amplification and sequencing of DNA from environmental samples. N. Z. J. Ecol..

[39] Callahan B.J., McMurdie P.J., Rosen M.J., Han A.W., Johnson A.J.A., Holmes S.P. (2016). DADA2: High-resolution sample inference from Illumina amplicon data. Nat. Methods.

[40] Martin M. (2011). Cutadapt removes adapter sequences from high-throughput sequencing reads. EMBnet J..

[41] Murali A., Bhargava A., Wright E.S. (2018). IDTAXA: A novel approach for accurate taxonomic classification of microbiome sequences. Microbiome.

[42] UNITE Community (2019). Full UNITE+INSD Dataset for Fungi, UNITE Community Version 18.11.2018.

[43] Zhou J., Jiang X., Zhou B., Zhao B., Ma M., Guan D., Li J., Chen S., Cao F., Shen D. (2016). Thirty four years of nitrogen fertilization decreases fungal diversity and alters fungal community composition in black soil in northeast China. Soil Biol. Biochem..

[44] Sun R., Dsouza M., Gilbert J.A., Guo X., Wang D., Guo Z., Ni Y., Chu H. (2016). Fungal community composition in soils subjected to long-term chemical fertilization is most influenced by the type of organic matter. Environ. Microbiol..

[45] Ye G., Lin Y., Luo J., Di H.J., Lindsey S., Liu D., Fan J., Ding W. (2020). Responses of soil fungal diversity and community composition to long-term fertilization: Field experiment in an acidic Ultisol and literature synthesis. Appl. Soil Ecol..

[46] Xiong W., Li R., Ren Y., Liu C., Zhao Q., Wu H., Jousset A., Shen Q. (2017). Distinct roles for soil fungal and bacterial communities associated with the suppression of vanilla Fusarium wilt disease. Soil Biol. Biochem..

[47] Van Geel B., Buurman J., Brinkkemper O., Schelvis J., Aptroot A., Van Reenen G., Hakbijl T. (2003). Environmental reconstruction of a Roman Period settlement site in Uitgeest (The Netherlands), with special reference to coprophilous fungi. J. Archaeol. Sci..

[48] Semenov M.V., Nikitin D.A., Stepanov A.L., Semenov V.M. (2019). The structure of bacterial and fungal communities in the rhizosphere and root-free loci of gray forest soil. Eurasian Soil Sci..

[49] Mapperson R.R., Kotiw M., Davis R.A., Dearnaley J.D. (2014). The diversity and antimicrobial activity of Preussia sp. endophytes isolated from Australian dry rainforests. Curr. Microbiol..

[50] Philippot L., Raaijmakers J.M., Lemanceau P., Van Der Putten W.H. (2013). Going back to the roots: The microbial ecology of the rhizosphere. Nat. Rev. Microbiol..

[51] Ai C., Liang G., Sun J., Wang X., He P., Zhou W., He X. (2015). Reduced dependence of rhizosphere microbiome on plant-derived carbon in 32-year long-term inorganic and organic fertilized soils. Soil Biol. Biochem..

[52] Chen C., Zhang J., Lu M., Qin C., Chen Y., Yang L., Huang Q., Shen Z., Shen Q. (2016). Microbial communities of an arable soil treated for 8 years with organic and inorganic fertilizers. Biol. Fertil. Soils.

[53] Van Diepeningen A.D., De Vos O.J., Zelenev V.V., Semenov A.M., Van Bruggen A.H. (2005). DGGE fragments oscillate with or counter to fluctuations in cultivable bacteria along wheat roots. Microb. Ecol..

[54] Bender S.F., Wagg C., van der Heijden M.G. (2016). An underground revolution: Biodiversity and soil ecological engineering for agricultural sustainability. Trends Ecol. Evol..

[55] Van Bruggen A.H.C., Finckh M.R. (2016). Plant diseases and management approaches in organic farming systems. Annu. Rev. Phytopathol..

[56] Berendsen R.L., Pieterse C.M., Bakker P.A. (2012). The rhizosphere microbiome and plant health. Trends Plant Sci..

[57] Lewis J.A., Fravel D.R., Papavizas G.C. (1995). *Cladorrhinum foecundissimum*: A potential biological control agent for the reduction of *Rhizoctonia solani*. Soil Biol. Biochem..

[58] van Bruggen A.H., Sharma K., Kaku E., Karfopoulos S., Zelenev V.V., Blok W.J. (2015). Soil health indicators and Fusarium wilt suppression in organically and conventionally managed greenhouse soils. Appl. Soil Ecol..

[59] van Bruggen A.H., Narouei-Khandan H.A., Gravel V., Blok W.J. (2016). Corky root severity, root knot nematode galling and microbial communities in soil, rhizosphere and rhizoplane in organic and conventional greenhouse compartments. Appl. Soil Ecol..

[60] Banerjee S., Walder F., Büchi L., Meyer M., Held A.Y., Gattinger A., Keller T., Charles R., van der Heijden M.G. (2019). Agricultural intensification reduces microbial network complexity and the abundance of keystone taxa in roots. ISME J..

[61] Berg M., Koskella B. (2018). Nutrient-and dose-dependent microbiome-mediated protection against a plant pathogen. Curr. Biol..

[62] Vinale F., Sivasithamparam K., Ghisalberti E.L., Marra R., Woo S.L., Lorito M. (2008). Trichoderma–plant–pathogen interactions. Soil Biol. Biochem..

[63] Ko W.H., Yang C.H., Lin M.J., Chen C.Y., Tsou Y.J. (2011). *Humicola phialophoroides* sp. nov. from soil with potential for biological control of plant diseases. Bot. Stud..

[64] Suleiman A.K., Harkes P., van den Elsen S., Holterman M., Korthals G.W., Helder J., Kuramae E.E. (2019). Organic amendment strengthens interkingdom associations in the soil and rhizosphere of barley (*Hordeum vulgare*). Sci. Total Environ..

[65] A’Bear A.D., Jones T.H., Boddy L. (2014). Potential impacts of climate change on interactions among saprotrophic cord-forming fungal mycelia and grazing soil invertebrates. Fungal Ecol..

[66] Goncharov A.A., Glebova A.A., Tiunov A.V. (2020). Trophic interactions between Fusarium species and soil fauna: A meta-analysis of experimental studies. Appl. Soil Ecol..

