# Supplementary material for: Mineral and Organic Fertilizers Distinctly Affect Fungal Communities in the Crop Rhizosphere
Source: J Fungi (Basel). 2022 Mar 1;8(3):251. doi: 10.3390/jof8030251 (PMC8949291; doi:10.3390/jof8030251)
Supplement: Supplementary file 1 [file jof-08-00251-s001.zip › jof-1616568-supplementary.pdf]

SUPPLEMENTARY FIGURES

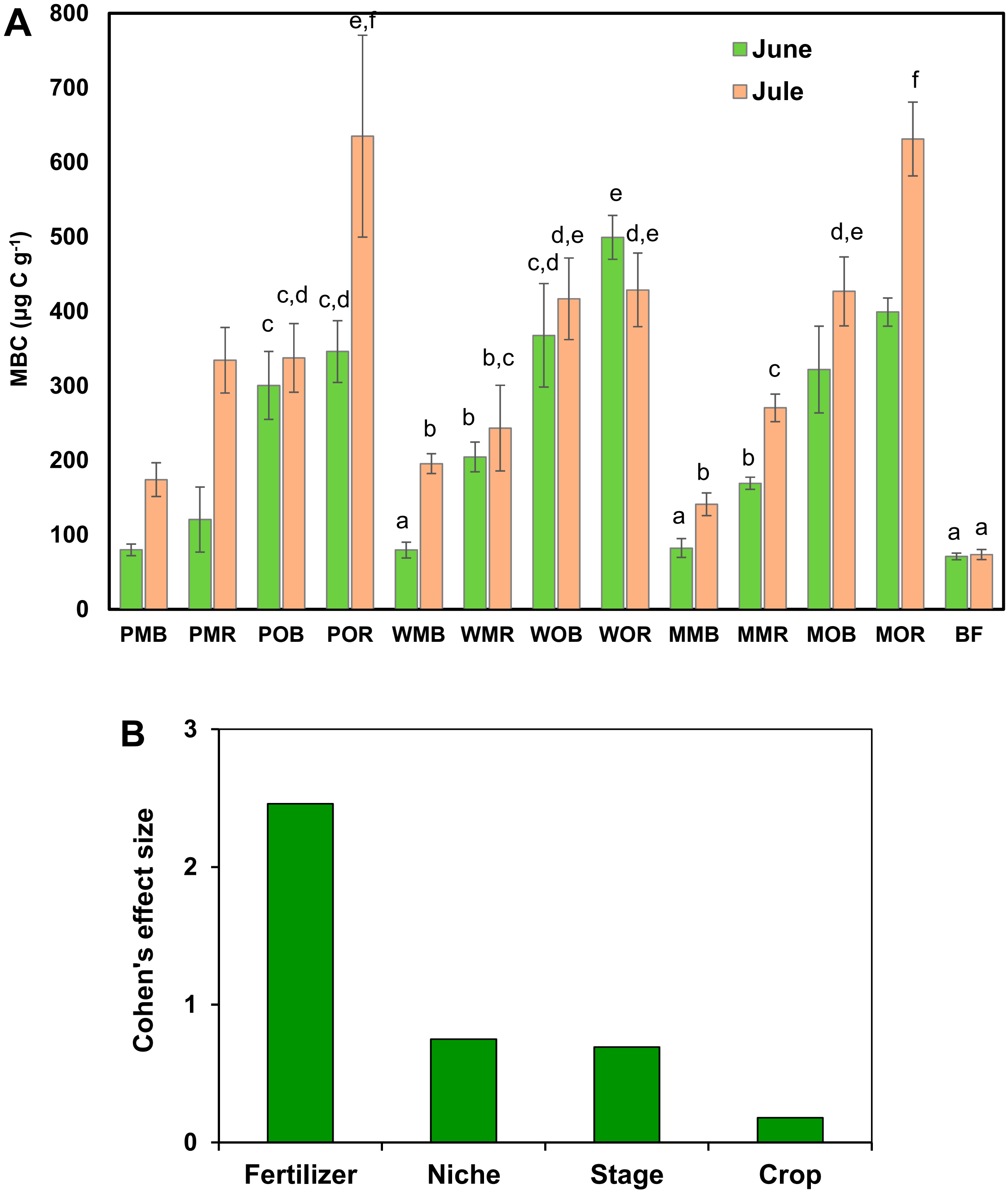

Figure S1. (A) Microbial biomass carbon (MBC) of the studied samples. All soil samples are indexed according to the scheme: «plant–fertilizer system–soil niche–sampling period». The following indices were used for (I) the crop species: P— potato, W — white mustard, M — maize; (II) fertilizer systems: M — mineral and O — organic; (III) soil niches: B— bulk soil, R— rhizosphere. The bare fallow without fertilizers is marked as BF. (B) The contribution of the ecological factors (niche, fertilizer system, crop type, stage of plant development) into MBC estimated using Cohen's *d* effect size.

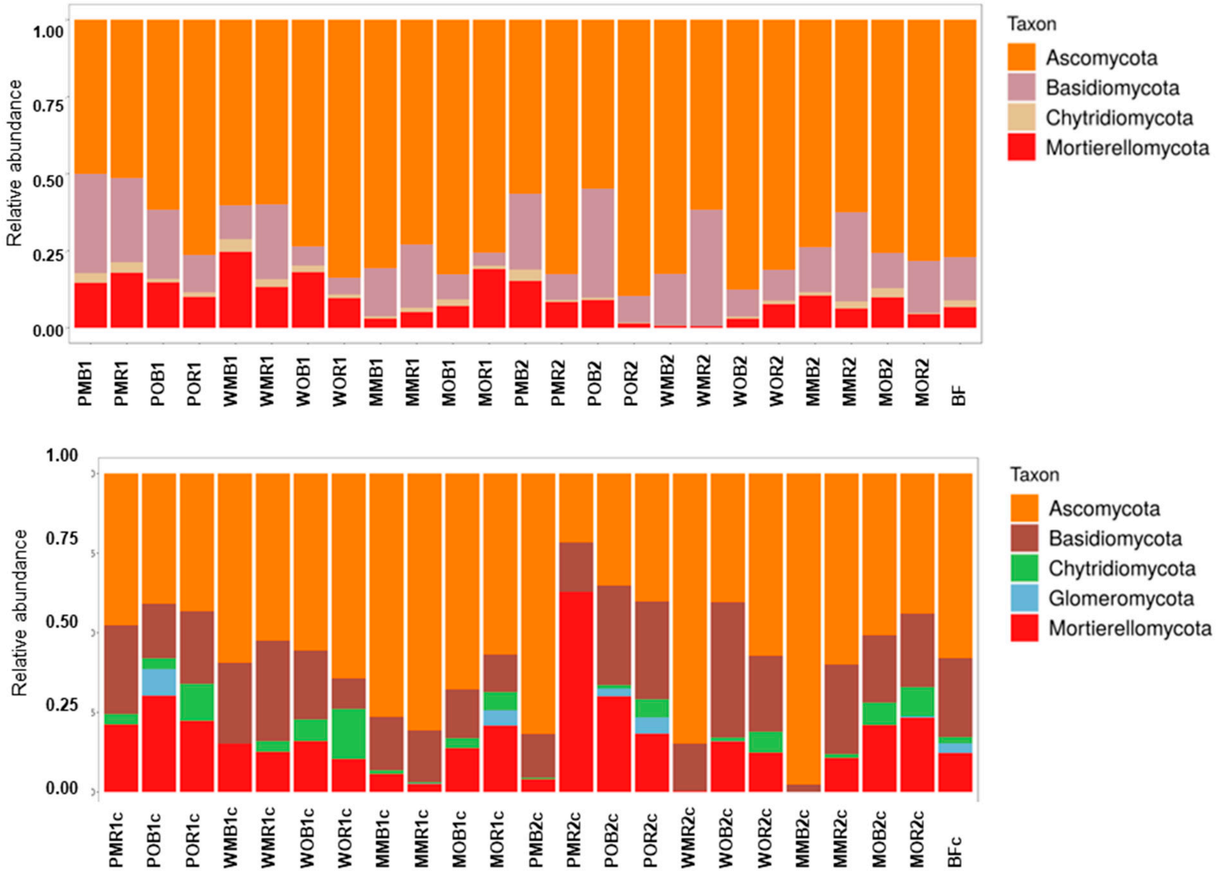

Figure S2. The structure of total (upper) and active (lower) fungal communities in studied samples at phylum level (the data are presented for taxa with abundance more than 0.5%). All soil samples are indexed according to the scheme: «plant–fertilizer system–soil niche–sampling period». The following indices were used for (I) the crop species: P— potato, W — white mustard, M — maize; (II) fertilizer systems: M — mineral and O — organic; (III) soil niches: B— bulk soil, R— rhizosphere; (IV) periods of sampling: 1 — June, 2 — July. The bare fallow without fertilizers is marked as BF.

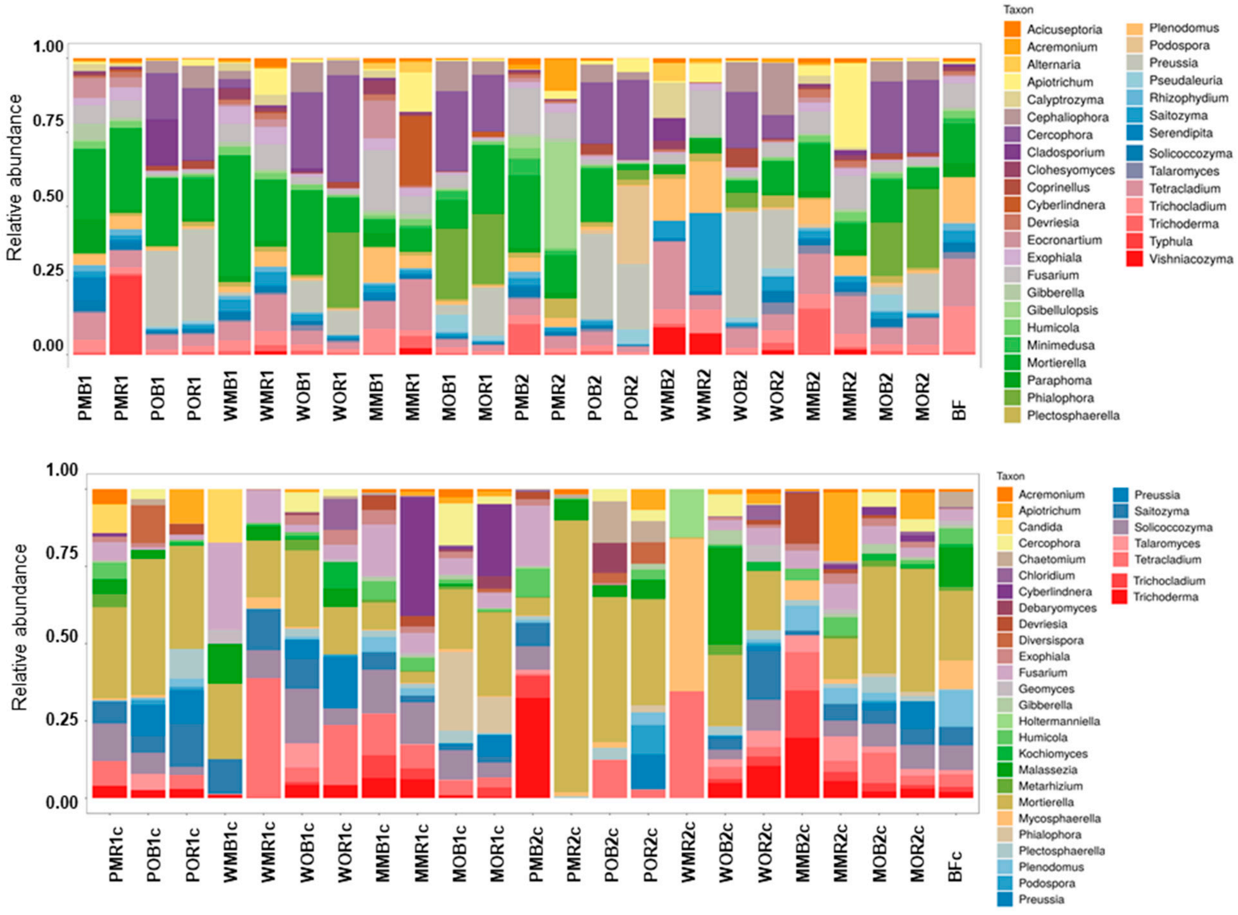

Figure S3. The structure of total (upper) and active (lower) fungal communities in studied samples at genera level (the data are presented for taxa with abundance more than 0.5%). All soil samples are indexed according to the scheme: «plant–fertilizer system–soil niche–sampling period». The following indices were used for (I) the crop species: P— potato, W — white mustard, M — maize; (II) fertilizer systems: M — mineral and O — organic; (III) soil niches: B— bulk soil, R— rhizosphere; (IV) periods of sampling: 1 — June, 2 — July. The bare fallow without fertilizers is marked as BF.

SUPPLEMENTARY TABLES

Table S1. Basic chemical characteristics of the studied soil and rhizosphere samples collected in June and July of 2017 from a long-term fertilization experiment in Pushchino, Russia.

Index	Plant	Fertilizer system	Niche	TOC, %	TN, %	C/N	pH $_{H2O}$
			June				
PMB-1	Potato	Mineral	Soil	1.23	0.13	9.4	4.51
PMR-1			Rhizosphere	1.34	0.13	10.0	4.53
POB-1		Organic	Soil	1.68	0.15	11.0	5.92
POR-1			Rhizosphere	1.80	0.16	11.5	6.11
WMB-1	White mustard	Mineral	Soil	1.23	0.13	9.4	4.33
WMR-1			Rhizosphere	1.36	0.14	9.7	4.82
WOB-1		Organic	Soil	1.59	0.15	10.4	6.27
WOR-1			Rhizosphere	1.76	0.16	11.0	6.37
MMB-1	Maize	Mineral	Soil	1.22	0.13	9.0	4.44
MMR-1			Rhizosphere	1.35	0.14	9.5	4.56
MOB-1		Organic	Soil	1.67	0.16	10.6	5.75
MOR-1			Rhizosphere	1.81	0.17	10.9	6.20
			July				
PMB-2	Potato	Mineral	Soil	1.22	0.12	10.0	4.39
PMR-2			Rhizosphere	1.35	0.13	10.3	4.41
POB-2		Organic	Soil	1.64	0.16	10.4	6.16
POR-2			Rhizosphere	1.97	0.19	10.4	6.39
WMB-2	White mustard	Mineral	Soil	1.22	0.13	9.3	4.75
WMR-2			Rhizosphere	1.28	0.13	9.6	4.76
WOB-2		Organic	Soil	1.64	0.16	10.3	6.16
WOR-2			Rhizosphere	1.89	0.17	11.3	6.19
MMB-2	Maize	Mineral	Soil	1.21	0.12	9.8	4.40
MMR-2			Rhizosphere	1.33	0.13	10.2	4.58
MOB-2		Organic	Soil	1.58	0.15	10.3	6.15
MOR-2			Rhizosphere	1.82	0.17	10.8	6.35
BF	Bare fallow		Soil	1.01	0.11	9.1	6.12

Table S2. The contribution of main and interaction effects among the ecological factors (niche, fertilizer system, crop type, stage of plant development) onto dispersion of MBC and fungal gene copies abundances using N-way ANOVA. Only statistically significant ($p < 0.05$) factors were used.

**Treatment**	**F**	**ή²**
MBC		
Fertilizer	302	0.61
Niche	61.25	0.12
Stage	53.01	0.11
Crop × Niche × Stage	6.63	0.03
Fungal abundance		
Fertilizer	918.71	0.57
Niche	353.46	0.10
Stage	162.53	0.22
Fertilizer × Stage	20.31	0.01
Fertilizer × Crop	19.39	0.02
Fertilizer × Crop × Stage	18.62	0.01
Niche × Stage	10.67	0.01
Crop × Stage	8.64	0.01
Crop	6.24	0.01
All 4 factors	7.6	0.01

Table S3. The lack of overall response of the most abundant fungal phyla to fertilization (Organic vs. Mineral) in bulk soil and rhizosphere.

Taxon	**t-test p-value**	**Mann-Whitney U-test p-value**	**Spearman r**
Bulk soil			
Glomeromycota	0.3	0.5	0.13
Olpidiomycota	0.07	0.01	0.51
Basidiomycota	0.8	0.2	-0.25
Mortierellomycota	0.7	0.6	0.10
Ascomycota	0.7	0.4	0.16
Monoblepharomycota	0.8	0.07	-0.36
Chytridiomycota	0.9	0.9	0.02
Mucoromycota	0.2	0.2	0.29
Entomophthoromycota	0.3	0.3	-0.20
Rozellomycota	0.9	1.0	-0.01
Zoopagomycota	0.3	0.3	-0.20
Kickxellomycota	0.4	0.8	-0.05
Rhizosphere			
Basidiomycota	0.002	0.003	-0.59
Ascomycota	0.007	0.007	0.54
Chytridiomycota	0.10	0.2	-0.30
Olpidiomycota	0.07	0.002	0.64
Monoblepharomycota	0.05	0.001	-0.68
Mortierellomycota	0.8	0.5	-0.16
Mucoromycota	0.08	0.04	-0.43
Rozellomycota	0.9	0.3	-0.22
Aphelidiomycota	0.3	0.4	0.20
Entomophthoromycota	0.3	0.3	-0.21
Zoopagomycota	0.3	0.3	-0.21
Kickxellomycota	0.3	0.8	-0.05
Glomeromycota	1.0	0.8	0.05

Table S4. The top 70 fungal genera responsive to fertilization (Organic vs. Mineral) in bulk soil and rhizosphere (potentially plant pathogenic genera in **bold**).

Taxon	**t-test p-value**	**Mann-Whitney U-test p-value**	**Spearman r**
Bulk soil			
Cercophora	0.0003	0.0001	0.76
Preussia	0.006	0.0001	0.76
Cephaliophora	0.0005	0.0003	0.71
Inocybe	0.0007	0.0001	0.77
Plenodomus	0.001	<0.0001	-0.73
Devriesia	0.0003	0.0001	-0.75
Ascobolus	0.0010	0.002	0.62
Calyptrozyma	0.03	0.002	-0.61
Clohesyomyces	0.01	0.001	-0.64
Fusarium	0.006	0.0004	-0.66
Thermomyces	0.0003	0.0005	0.69
Paraphoma	0.03	0.0003	-0.72
Furcaspora	0.02	<0.0001	-0.77
Trichocladium	0.007	0.0008	-0.63
Ophiosphaerella	0.010	0.001	-0.64
Phialophora	0.04	0.010	0.51
Exophiala	0.009	0.003	-0.57
Conlarium	0.005	0.0008	-0.66
Drechslera	0.01	0.0006	-0.68
Geomyces	0.0005	0.0003	-0.72
Setophoma	0.005	0.0003	-0.72
Pseudaleuria	0.05	0.005	0.56
Meliniomyces	0.03	0.002	-0.61
Neobulgaria	0.005	0.003	-0.58
Serendipita	0.06	<0.0001	-0.89
Rhizophlyctis	0.03	0.004	0.58
Metacordyceps	0.0006	0.005	-0.55
Ganoderma	0.007	0.007	-0.53
Lectera	0.001	0.0004	-0.70
Oidiodendron	0.03	0.004	-0.56
Scedosporium	0.008	0.001	0.63
Spiromastix	0.01	0.01	-0.50
Tetracladium	0.05	0.003	-0.56
Mycothermus	0.003	0.0003	0.71
Pseudosigmoidea	0.01	0.05	-0.39
Conocybe	0.08	0.04	0.40
Eocronartium	0.1	0.005	-0.56
Cladorrhinum	0.06	0.05	0.40
Clavispora	0.1	0.006	-0.55
Coniochaeta	0.08	0.004	0.57
Paraphaeosphaeria	0.09	0.07	-0.36
Cladophialophora	0.007	0.0003	-0.71

Echinoderma	0.02	0.0009	-0.66
Schwanniomyces	0.02	0.002	-0.61
Scytalidium	0.02	0.02	0.45
Lycoperdon	0.008	0.01	-0.49
Arthrobotrys nematodes	0.02	0.002	-0.60
Trematosphaeria	0.07	0.2	-0.26
Vishniacozyma	0.1	0.0010	-0.65
Penicillium	0.02	0.01	-0.49
Truncatella	0.02	0.01	0.49
Trichoderma	0.1	0.3	-0.23
Coprinus	0.04	0.002	0.60
Gibellulopsis	0.1	0.03	-0.43
Athelopsis	0.2	0.02	-0.45
Coprinellus	0.06	1.0	0.01
Monodictys	0.1	0.09	0.34
Saitozyma	0.1	0.02	-0.47
Acicuseptoria	0.1	0.02	-0.45
Simocybe	0.04	0.002	0.61
Gibberella	0.1	0.03	-0.42
Chloridium	0.08	0.04	0.40
Humicola	0.1	0.09	-0.33
Microascus	0.02	0.01	0.51
Diversispora	0.3	0.09	0.34
Cladosporium	0.6	0.01	-0.50
Corticium	0.05	0.01	0.51
Botrytis	0.2	0.04	-0.41
Acaulium	0.05	0.02	0.46
Microdochium	0.3	0.002	-0.61
Rhizosphere			
Cercophora	<0.0001	<0.0001	0.88
Preussia	0.0004	<0.0001	0.81
Cephaliophora	0.007	<0.0001	0.81
Exophiala	<0.0001	<0.0001	-0.87
Phialophora	0.02	0.0009	0.68
Plenodomus	0.001	<0.0001	-0.87
Fusarium	<0.0001	<0.0001	-0.85
Ascobolus	0.0007	<0.0001	0.84
Thermomyces	<0.0001	<0.0001	0.92
Serendipita	0.002	<0.0001	-0.82
Devriesia	0.002	0.0007	-0.69
Pseudaleuria	0.03	0.002	0.64
Gibberella	0.0009	<0.0001	-0.74
Inocybe	0.008	<0.0001	0.84
Acicuseptoria	0.005	0.0010	-0.68
Calyptrozyma	0.02	0.005	-0.58
Paraphoma	0.006	0.002	-0.64
Eocronartium	0.02	0.009	-0.54
Ophiosphaerella	0.009	0.0006	-0.71

Apiotrichum	0.03	0.05	-0.41
Venturia	0.002	0.0003	-0.75
Mycosphaerella	0.007	0.006	-0.56
Microdochium	0.002	0.0003	-0.74
Clohesyomyces	0.01	0.01	-0.52
Athelopsis	0.03	<0.0001	-0.81
Cladorrhinum	0.02	0.02	0.48
Conlarium	0.010	<0.0001	-0.86
Drechslera	0.008	0.0006	-0.71
Rhizophydium	0.02	0.06	-0.38
Meliniomyces	0.06	0.0005	-0.71
Metacordyceps	0.003	0.002	-0.62
Pyrenochaeta	0.04	0.2	-0.29
Furcaspora	0.07	<0.0001	-0.87
Operculomyces	0.02	0.0007	0.70
Boeremia	0.04	0.02	-0.46
Humicola	0.06	0.03	-0.44
Tetracladium	0.06	0.03	-0.43
Holtermanniella	0.07	0.01	-0.53
Scedosporium	0.008	0.0007	0.70
Clavispora	0.07	0.0007	-0.70
Rhizophlyctis	0.04	0.03	0.45
Cryptococcus	0.009	0.005	-0.58
Conocybe	0.06	0.1	0.32
Botrytis	0.10	0.0003	-0.74
Lectera	0.09	0.0001	-0.78
Vishniacozyma	0.09	0.008	-0.55
Setophoma	0.1	<0.0001	-0.81
Acremonium	0.1	0.0002	-0.76
Bullera	0.06	0.008	-0.55
Gibellulopsis	0.2	0.03	-0.44
Mycothermus	0.02	0.004	0.59
Podospora	0.2	0.0004	0.72
Cotylidia	0.08	0.0002	-0.76
Cladophialophora	0.01	0.0005	-0.71
Typhula	0.2	0.1	-0.31
Cladosporium	0.1	0.001	-0.67
Clonostachys	0.1	0.09	-0.35
Coprinus	0.03	0.0007	0.70
Acaulium	0.01	0.004	0.59
Olpidium	0.07	0.002	0.64
Dendryphion	0.02	0.005	-0.58
Simocybe	0.02	0.010	0.53
Ganoderma	0.06	0.09	-0.35
Udeniozyma	0.07	0.10	-0.34
Kotlabaea	0.05	0.05	-0.40
Fusicolla	0.2	0.03	-0.44
Truncatella	0.03	0.03	0.45

Leucosporidium	0.06	0.05	-0.41
Ustilentyloma	0.01	0.005	-0.58
Kochiomyces	0.2	0.05	-0.41

Table S5. Fungal taxa associated with rhizosphere of potato, white mustard and maize. Genera with a share of >0.1%, which relative abundance increased by at least 10 times for each of the samples as a result of any of the treatments.

**Potato**	**Mustard**	**Maize**	**Potato &Mustard**	**Potato & Maize**	**Maize & Mustard**
Clonostachys	*Ceratobasidium*	*Candida*	*Acremonium*	*Acaulium*	*Athelopsis*
Colletotrichum	*Clitopilus*	*Conocybe*	*Coprinopsis*	*Acidomelania*	*Bullera*
Cotylidia	*Filobasidium*	*Cutaneotrichosporon*	*Operculomyces*	*Arthrobotrys*	*Crocicreas*
Gibellulopsis	*Fomitopsis*	*Cyberlindnera*		*Chaetomidium*	*Holtermanniella*
Heterogastridium	*Glarea*	*Mycena*		*Clavispora*	*Kernia*
Hymenoscyphus	*Micarea*	*Scutellinia*		*Coniochaeta*	*Monodictys*
Incertomyces	*Occultifur*	*Vermispora*		*Fusicolla*	*Phialophora*
Pilidium	*Panaeolus*			*Lectera*	*Rhynchogastrema*
Podospora	*Trechispora*			*Mycothermus*	*Vishniacozyma*

Table S6. Fungal taxa associated with organic or mineral fertilizers. Genera with a share of >0.1%, which relative abundance increased by at least 10 times for each of the samples after fertilization by manure or mineral fertilizer.

**Activation by NPK**	**Activation by manure**	**Activation by NPK & manure**	**Suppression by NPK**	**Suppression by manure**
Acremonium	*Ascobolus*	*Acidomelania*	*Agrocybe*	*Cladophialophora*
Athelopsis	*Cephaliophora*	*Apiotrichum*	*Caryophylloseptoria*	*Cladosporium*
Botrytis	*Cercophora*	*Arthrobotrys*	*Coprinus*	*Clitopilus*
Bullera	*Cladorrhinum*	*Boeremia*	*Cortinarius*	*Conlarium*
Calyptrozyma	*Coniochaeta*	*Chaetomidium*	*Gamsia*	*Emericellopsis*
Candida	*Conocybe*	*Cheilymenia*	*Pluteus*	*Eocronartium*
Ceratobasidium	*Coprinopsis*	*Clonostachys*	*Trichophyton*	*Furcaspora*
Clavispora	*Heterogastridium*	*Colletotrichum*		*Gregorella*
Cotylidia	*Inocybe*	*Coprinellus*		*Gymnoascus*
Cryptococcus	*Monodictys*	*Cyberlindnera*		*Gymnopilus*
Cutaneotrichosporon	*Mycothermus*	*Dendryphion*		*Helicodendron*
Filobasidium	*Olpidium*	*Dioszegia*		*Knufia*
Fomitopsis	*Operculomyces*	*Itersonilia*		*Kochiomyces*
Fusicolla	*Panaeolus*	*Mycosphaerella*		*Kotlabaea*
Gibellulopsis	*Phialophora*	*Plectosphaerella*		*Leptospora*
Glarea	*Pilidium*	*Pleurophragmium*		*Meliniomyces*
Holtermanniella	*Podospora*	*Psathyrella*		*Microdochium*
Incertomyces	*Preussia*	*Pyrenochaetopsis*		*Neobulgaria*
Lectera	*Pseudaleuria*	*Rhizophlyctis*		*Nigrospora*
Micarea	*Scedosporium*	*Scutellinia*		*Oidiodendron*
Mycena	*Thermomyces*	*Vishniacozyma*		*Ophiosphaerella*
Rhynchogastrema	*Trechispora*			*Periconia*
Rhynchosporium				*Plenodomus*
Setophoma				*Pyrenochaeta*
Tricholoma				*Sagenomella*
Typhula				*Sakaguchia*
Vermispora				*Serendipita*
				Spirosphaera
				Urnula
				Venturia

Table S7. PERMANOVA and ANOSIM tests of variance and similarities of total fungal communities' using Bray-Curtis (BC) distance matrix.

Treatment	Groups	PERMANOVA		ANOSIM	
		Pseudo-F	p-value	R	p-value
Bulk soil and rhizosphere; Total fungal communities (N = 52)					
Fertilizer	3	12.98	0.001	0.877	0.001
Plant	4	1.72	0.026	0.053	0.084
Niche	4	1.05	0.357	0.033	0.14
Stage	2	0.983	0.40	-0.032	0.928
Fertilizer × Plant	7	6.63	0.001	0.678	0.001
Fertilizer × Niche	5	7.29	0.001	0.634	0.001
Fertilizer × Stage	5	7.11	0.001	0.609	0.001
Plant × Niche	7	1.344	0.064	0.039	0.168
Plant × Stage	7	1.292	0.109	0.018	0.313
Niche × Stage	4	1.013	0.398	0.609	0.232
Only rhizosphere; Total fungal communities (N = 25)					
Fertilizer	2	13.52	0.001	0.953	0.001
Plant	3	1.31	0.179	0.036	0.227
Stage	2	1.16	0.273	-0.022	0.559
Fertilizer × Plant	6	4.93	0.001	0.707	0.001
Fertilizer × Stage	4	5.82	0.001	0.646	0.001
Plant × Stage	6	1.11	0.313	0.002	0.446
